# Recanalization of XEN-45 gel stent occlusion with cortical material after phaco-XEN surgery using Nd: YAG laser treatment: A case report

**DOI:** 10.1097/MD.0000000000027010

**Published:** 2021-08-27

**Authors:** Je Hyun Seo, Su-Ho Lim

**Affiliations:** a Veterans Medical Research Institute, Veterans Health Service Medical Center, South Korea; b Department of Ophthalmology, Daegu Veterans Health Service Medical Center, Daegu, South Korea.

**Keywords:** cortical material, Glaucoma, neodymium-doped yttrium aluminum garnet laser, XEN gel stent, XEN occlusion

## Abstract

**Introduction:**

The XEN Gel Stent (Allergan Inc., CA, USA) has been widely used in minimally invasive glaucoma surgery to lower intraocular pressure considering reasonable efficacy and safety profiles. However, the XEN gel stent could be occluded by fibrin, blood clots, or the iris due to its small lumen design for preventing postoperative hypotony. To date, only a few studies have assessed XEN occlusion after combined phacoemulsification with XEN gel implantation and how to manage this condition. We describe the first case report of XEN gel stent obstruction by cortical material, demonstrated by anterior segment optical coherence tomography (AS-OCT), which resolved effectively after low-energy neodymium-doped yttrium aluminum garnet (Nd: YAG) laser shock wave treatment.

**Patient Information:**

A 76-year-old Korean male patient underwent uncomplicated phaco-XEN-gel stent implantation and presented with low intraocular pressure (IOP) with a well-functioning filtering bleb during the first postoperative 4 days. On postoperative day 5, the XEN lumen was occluded by the cortical material, with an intraocular pressure elevation of 28 mm Hg. Slit-lamp examination revealed that cortical material was causing a block into the internal ostium of the XEN gel implant. AS-OCT examination also demonstrated the presence of hyper-reflective materials at the intraluminal portion and peritubular portion around the internal ostium of the XEN gel implant.

**Diagnosis:**

XEN gel stent occlusion with cortical material after Phaco-XEN surgery.

**Interventions:**

Low-energy Nd: YAG laser shock wave treatment.

**Outcomes:**

The IOP dropped from 28 mm Hg to 8 mm Hg immediately after treatment. Transient hypotony and a slightly shallow anterior chamber were noted over the 3 days after YAG laser treatment. The IOP continued to be well-controlled until 3 months later (range; 6 - 12 mm Hg).

**Conclusions:**

To the best of our knowledge, this is the first case report on the efficacy of Nd: YAG laser treatment for recanalization of XEN implant occluded by the cortex. Moreover, AS-OCT could provide additional clinical information for recanalization of the XEN gel stent.

## 1 Introduction

Glaucoma is a chronic, progressive optic neuropathy with characteristic changes in the optic nerve head and visual field and is one of the leading causes of blindness worldwide.^[1,2]^ Elevated intraocular pressure (IOP) is a major risk factor for the development and progression of glaucoma.^[1–3]^ Medical management is often insufficient, especially in advanced disease stages, and surgical intervention is required.^[3]^ Minimally invasive glaucoma surgery has recently been widely used to control elevated IOP.^[4,5]^

XEN GEL Stent (Allergan Inc., CA, USA) is a 6-mm hydrophilic tube of collagen-derived gelatin that is cross-linked with glutaraldehyde.^[4,5]^ Xen Gel Stent have demonstrated an IOP-lowering effect via subconjunctival drainage without tissue disruption compared to trabeculectomy or traditional glaucoma drainage implants.^[4,5]^ XEN 45 has been designed to prevent early postoperative hypotony; it has a small lumen with an inner opening of 45 μm. Thus, the design has an inherent probability of being occluded by fibrin, blood clots, or the iris.^[6–9]^ However, to date, only a few studies have assessed XEN occlusion and its management. Herein, we describe the first case report of XEN gel stent obstruction by suspected “cortical materials” on anterior segment optical coherence tomography after combined phacoemulsification and XEN implantation, which resolved effectively after treatment with low-energy neodymium-doped yttrium aluminum garnet (Nd: YAG) laser shock wave therapy.

## 2 Case report

The study adhered to the principles of the Declaration of Helsinki, and written consent for the report and photographs was obtained from the patient. This case study was approved by the Institutional Review Board of Daegu Veterans Health Service Medical Center.

### 2.1 Initial presentation

**Figure 1:**
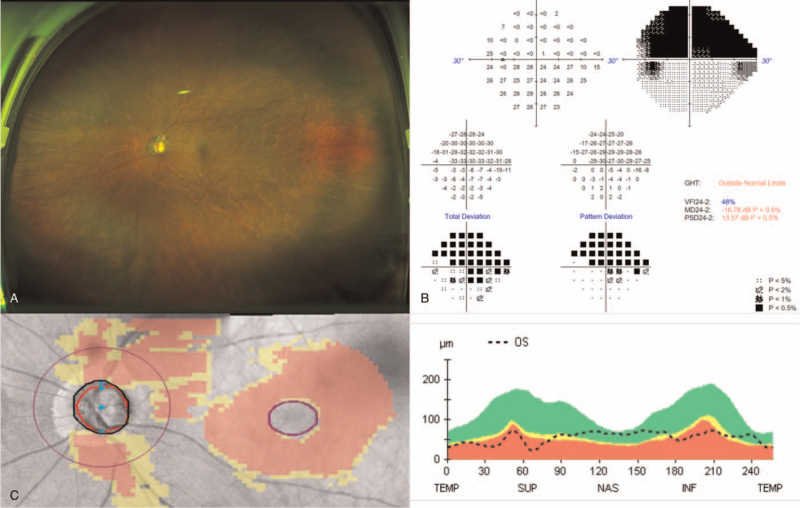
Preoperative ultra-widefield fundus photograph, visual field, and optical coherence tomography.

A 76-year-old Korean male patient was diagnosed with advanced primary open-angle glaucoma in both eyes, as well as systemic hypertension. Six months prior to presentation, the patient had undergone uneventful combined phacoemulsification and iStent Inject (Glaukos, CA, USA) surgery in his right eye. After the procedure, he had been using topical medical treatment, including a combination of dorzolamide and timolol (Cosopt, Santen, Japan), brimonidine tartrate 0.15% (Alphagan, Allergan, USA), and latanoprost (Xalatan, Pfizer, USA), in his left eye. At the time of presentation, his best-corrected visual acuity was 20/20 in his right eye, 20/100 in his left eye. And intraocular pressure (IOP) was 15 mm Hg in the right eye, 27 mm Hg in the left eye with the prescribed medication. Slit-lamp examination revealed a deep and quiet anterior chamber and nucleosclerosis and cortical opacities, suggesting senile cataract. Ultrawide-field fundus photography revealed a cup-to-disc ratio of 0.9, with inferior notching, corresponding to retinal nerve fiber layer (RNFL) thinning, laminar dot sign, and a slightly pale optic disc in the left eye. Visual field test and spectral-domain optical coherence tomography showed a superior hemifield defect (mean deviation: - 16.78 dB, visual field index: 48%) and reduced RNFL thickness on TSNIT graph (average RNFL thickness 59 μm) (Fig. 1). Gonioscopy revealed a wide iridocorneal angle (D40r; Spaeth classification), and brain magnetic resonance imaging was unremarkable.

### 2.2 Surgical procedure for phaco-XEN gel stent implantation

**Figure 2:**
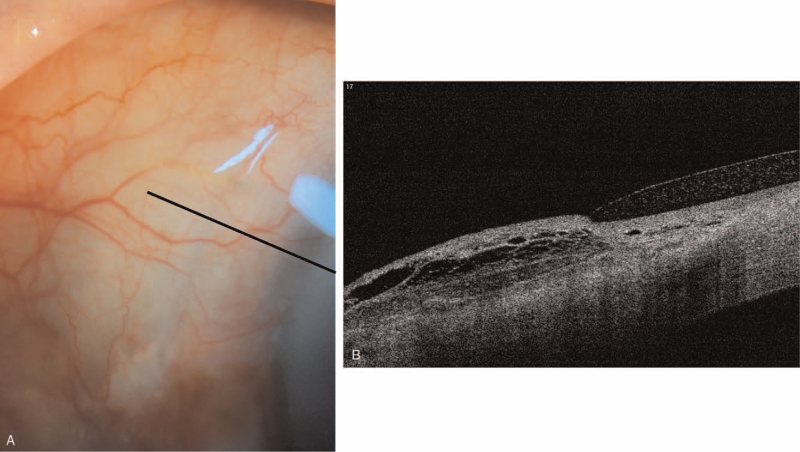
Anterior segment photography (A) and anterior segment optical coherence tomography (B) on postoperative day 3. A relatively well-functioning bleb and sponge-form hypo-reflective bleb were observed. (Black line: scan line).

Combined phacoemulsification and ab interno XEN gel stent implantation was performed. Briefly, under topical anesthesia, a 2.75-mm temporal clear corneal incision was made, and a side-port incision was made at 6 o’clock. Phacoemulsification with intraocular lens implantation was performed per standard procedures. A 0.1 mL mitomycin-C (0.4 mg/mL) was injected with a 30-gauze needle in the supero-nasal quadrant. The intended area of placement in the supero-nasal quadrant, 3.0 mm from the limbus, was marked. Then, an ab-interno XEN gel stent was implanted using mirrored goniolens (1.0 mm in the anterior chamber, 2.0 mm tunneled through the sclera, and 3.0 mm in subconjunctival space). The injector was gently withdrawn, and XEN placement was confirmed. Then, the viscoelastic material was removed from the anterior chamber. Finally, we checked bleb morphology and function with a forced infusion of a balanced salt solution. The patient was started on levofloxacin and prednisolone acetate 4 times daily on the next day. The IOP was 5 mm Hg on day 1 and 7 mm Hg on day 4 with a well-functioning bleb (Fig. 2).

### 2.3 XEN occlusion by cortex and neodymium-doped yttrium aluminum garnet laser treatment

**Figure 3:**
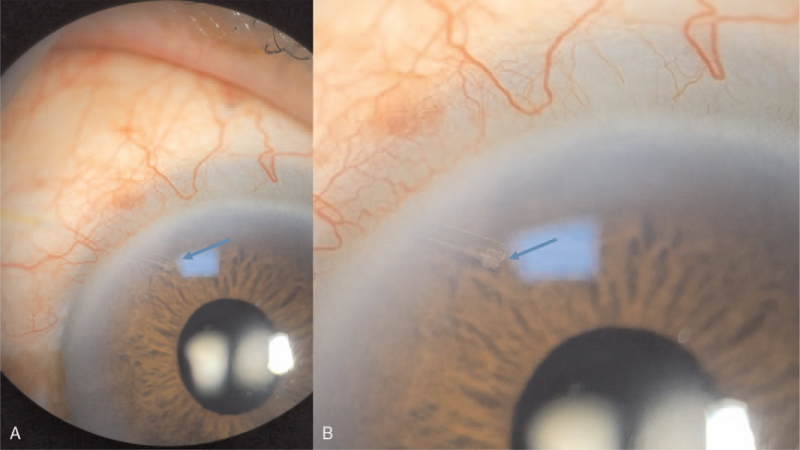
Anterior segment photography revealed that the suspected cortical materials were located at the peritubular portion and occluded the internal ostium of the XEN gel stent. These findings were noted at postoperative day 5 after combined phacoemulsification with XEN Gel Stent implantation surgery (A, Low magnification x 10, B, High magnification x 16).

**Figure 4:**
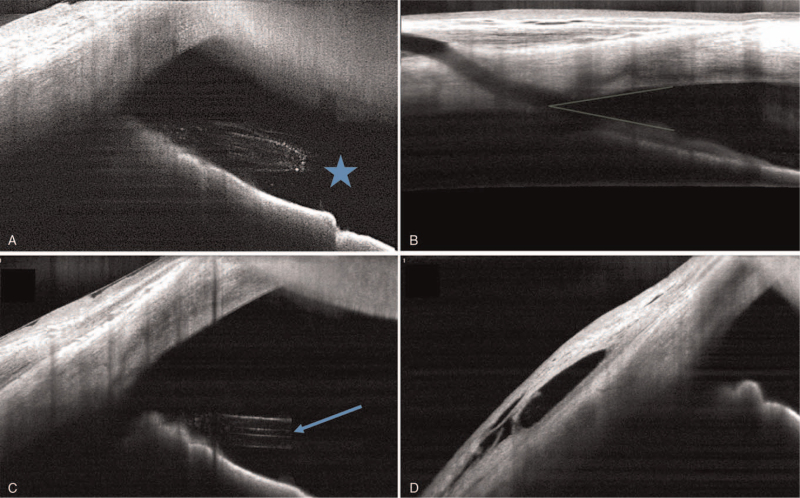
Anterior segment optical coherence tomography examination demonstrated the presence of hyper-reflective materials at the internal ostium of the XEN gel stent on postoperative day 5 (Blue star, A). Three days after laser treatment, transient hypotony and a slightly shallow anterior chamber were observed. (B) AS-OCT examination after 1 month confirmed the recanalization of the XEN gel stent (Blue arrow) and a functioning filtering bleb (C, D).

On follow-up observation, the patient's IOP spiked to 28 mm Hg on postoperative day 5. Slit-lamp examination showed that the tip of the XEN gel stent in the anterior chamber was occluded by suspected cortical materials and the presence of peritubular cortical material (Fig. 3). Anterior segment optical coherence tomography (AS-OCT) examination also revealed a “plug-in” shape and hyper-reflective materials near the inner opening of the stent (Fig. 4A).

### 2.4 Low-energy neodymium-doped yttrium aluminum garnet laser treatment and follow-up

**Figure 5:**
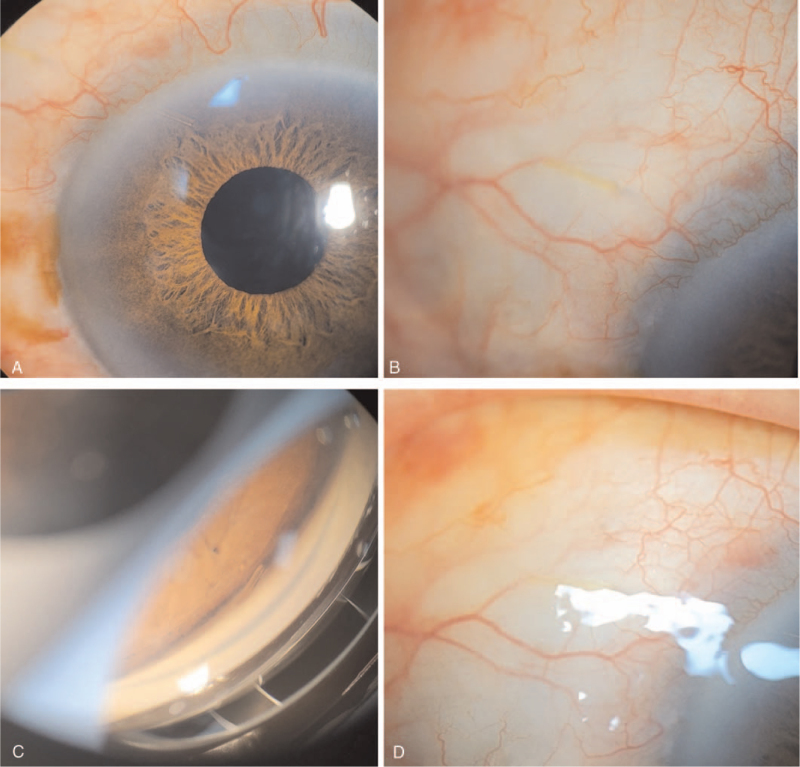
Anterior segment photography immediately post-laser treatment revealed the clearly visible inner opening and relatively well-functioning bleb (A, B). One week after laser treatment, a clearly visible inner opening of the XEN gel stent on gonioscopic examination (C) and functioning filtering bleb were observed on slit-lamp examination (D).

The authors performed low-energy neodymium-doped yttrium aluminum garnet (Nd: YAG) laser shockwave treatment with a peri-luminal anterior chamber tip under Goldmann 3 mirror gonioscopy to improve flow (0.8mJ, 2 shots). Immediately after laser treatment, the internal ostium was clearly visible (Fig. 5A, B), the IOP was substantially reduced to 8 mm Hg, and the bleb was formed at 2-hours post-laser treatment. Three days after laser treatment, transient hypotony and a slightly shallow anterior chamber were observed by slit-lamp examination and AS-OCT examination. (Fig. 4B).

One week after laser treatment, the patient's IOP stabilized to 9 mm Hg. Slit-lamp examination and gonioscopic examination showed a functioning filtering bleb with a clearly visible inner opening (Fig. 5C, D). Moreover, AS-OCT examination after 1 month confirmed the recanalization of the XEN gel stent and functioning filtering bleb (Fig. 4C, D). Follow-up through 3 months after laser treatment, the IOP was stable (range: 6–12 mm Hg) without other complication.

## 3 Discussion

The XEN45 gel stent has been recognized as a safe and effective minimally invasive glaucoma surgery procedure for advanced primary open angle glaucoma, exfoliative glaucoma, pigmentary glaucoma, and refractory glaucoma.^[4,5]^ Because of the small internal lumen (45 μm), reports have previously indicated that XEN occlusion can occur due to fibrin, blood clots, or the iris.^[6–10]^

This case report demonstrates that the cortical material after combined phacoemulsification with XEN implantation is a potential cause of XEN gel stent occlusion. To date, there have been no reports of early postoperative cortical material-XEN occlusion after combined surgery. Moreover, to the best of our knowledge, this is the first case report of internal ostium occlusion of a XEN implant, demonstrated by AS-OCT examination.

In this specific case, the exact nature of the whitish materials could not be clearly identified histologically. The possible materials are as follows:

1. blood clot,
2. fibrin plug,
3. cellular debris, or
4. cortical materials of a crystalline lens.

However, we hypothesized that cortical materials occluded the XEN gel implant based on the timing after surgery and the following reasons. Gillmann et al reported that anterior chamber RBCs can present a potential threat to XEN gel stent patency, even in cases when the bleeding was minimal and self-limited.^[7]^ A fibrin plug also is a possible material cause. Surgical stress-induced alteration of the blood-aqueous barrier might contribute to cellular debris-obstructing flow.^[7,8,10]^ However, there were no bleeding events or massive iris manipulation during XEN implantation in this patient. Moreover, there was no severe anterior chamber reaction during the follow-up period, and even a quiet cellular reaction at presentation (postoperative day 5). Thus, we concluded that the cortical material or hidden fine lens materials occluded the internal ostium because of the filtering effect from the anterior chamber to the subconjunctival space and local turbulence and pressure gradient along the tube.

Recently, Barao and colleagues reported that placement of the XEN stent in a more posterior location might be associated with increased early complications on an automated gonioscopic study. ^[11]^ In our patient, the approximate length of the XEN gel implant in the anterior chamber was 1.0 mm, and it was placed anteriorly to the scleral spur. We hypothesize that, for the patient in this report, there was enough space between the iris and the stent tip to prevent iris-XEN contact or iris-XEN occlusion in the early postoperative hypotony phase.

Adequate filtration of the aqueous humor is essential for successful XEN surgery. Thus, we decided to follow a “do-not-wait” approach for spontaneous reabsorption of cortical materials. Consequently, the authors anticipated a disruption effect of Nd: YAG laser treatment for cortical materials occluding the internal ostium of the XEN gel stent. Fellman et al. suggested hydrodynamic energy in the form of a shockwave that ravels down the lumen, dispersing and clearing invisible intraluminal cellular debris.^[12]^ After the Nd: YAG shock wave treatment, we re-established the flow of the cortical material-occluded XEN implant, which was similar to their study. After this approach, the patency of the XEN implant was improved dramatically. Thus, the authors suggest that ophthalmologists should consider Nd: YAG shock wave treatment prior to planning revision surgery.

In this patient, suspected cortical materials were found only surrounding the internal ostium on slit-lamp examination and AS-OCT. Rigo et al. reported hyper-reflective material on AS-OCT, whereas intraluminal obstruction was not visible using slit-lamp photography.^[13]^ Their report differed from the experience of our patient in visibility on slit-lamp examination. After laser treatment, patency was confirmed by both slit-lamp examination (inner opening, bleb height) and AS-OCT. As shown in our case, AS-OCT examination provides additional information about the position of the XEN implant, length of the protruded implant into the anterior chamber, or blockage of hyper-reflective material.

## 4 Conclusions and clinical significance

To the best of our knowledge, this is the first case report of recanalization using the Rescue Nd: YAG laser shock wave therapy for XEN-45 gel stent occlusion by suspected cortical materials after phaco-XEN surgery. Nd: YAG laser treatment might be a relatively safe and effective treatment for re-canalization of XEN occlusion after combined phacoemulsification with XEN gel implantation and surgery. AS-OCT examination might provide additional clinical information regarding the patency of XEN. Thus, ophthalmologists should consider Nd: YAG laser treatment and AS-OCT examination before planning surgical or medical treatment for XEN stent occlusion after combined Phaco-XEN surgery.

## Acknowledgments

The authors wish to thank eWorldEditing and PaperPal PreFlight service for language editing.

## Author contributions

**Conceptualization:** Je Hyun Seo, Su-Ho Lim.

**Data curation:** Je Hyun Seo, Su-Ho Lim.

**Formal analysis:** Je Hyun Seo, Su-Ho Lim.

**Funding acquisition:** Su-Ho Lim.

**Methodology:** Je Hyun Seo, Su-Ho Lim.

**Supervision:** Su-Ho Lim.

**Visualization:** Su-Ho Lim.

**Writing – original draft:** Je Hyun Seo, Su-Ho Lim.

**Writing – review & editing:** Je Hyun Seo, Su-Ho Lim.
